# Acquisition of the L452R Mutation in the ACE2-Binding Interface of Spike Protein Triggers Recent Massive Expansion of SARS-CoV-2 Variants

**DOI:** 10.1128/JCM.00921-21

**Published:** 2021-10-19

**Authors:** Veronika Tchesnokova, Hemantha Kulasekara, Lydia Larson, Victoria Bowers, Elena Rechkina, Dagmara Kisiela, Yulia Sledneva, Debarati Choudhury, Iryna Maslova, Kai Deng, Kirthi Kutumbaka, Hao Geng, Curtis Fowler, Dina Greene, James Ralston, Mansour Samadpour, Evgeni Sokurenko

**Affiliations:** a University of Washington, Seattle, Washington, USA; b ID Genomics, Inc., Seattle, Washington, USA; c IEH Laboratories and Consulting Group, Seattle, Washington, USA; d ARMADA (The Antibiotic Resistance Monitoring, Analysis and Diagnostics Alliance), Seattle, Washington, USA; e Kaiser Permanente Washington (KPWA), Seattle, Washington, USA; f KPWA Research Institute, Seattle, Washington, USA; University Hospital Münster

**Keywords:** L452R, SARS-CoV-2, expansion, mutation

## Abstract

We report that there is a recent global expansion of numerous independent severe acute respiratory syndrome coronavirus 2 (SARS-CoV-2) variants with mutation L452R in the receptor-binding domain (RBD) of the spike protein. The massive emergence of L452R variants was first linked to lineage B.1.427/B.1.429 (clade 21C) that has been spreading in California since November and December 2020, originally named CAL.20C and currently variant of interest epsilon. By PCR amplification and Sanger sequencing of a 541-base fragment coding for amino acids 414 to 583 of the RBD from a collection of clinical specimens, we identified a separate L452R variant that also recently emerged in California but derives from the lineage B.1.232, clade 20A (named CAL.20A). Notably, CAL.20A caused an infection in gorillas in the San Diego Zoo, reported in January 2021. Unlike the epsilon variant that carries two additional mutations in the N-terminal domain of spike protein, L452R is the only mutation found in the spike proteins of CAL.20A. Based on genome-wide phylogenetic analysis, emergence of both viral variants was specifically triggered by acquisition of L452R, suggesting a strong positive selection for this mutation. Global analysis revealed that L452R is nearly omnipresent in a dozen independently emerged lineages, including the most recent variants of concern/interest delta, kappa, epsilon and iota, with the lambda variant carrying L452Q. L452 is in immediate proximity to the angiotensin-converting enzyme 2 (ACE2) interaction interface of RBD. It was reported that the L452R mutation is associated with immune escape and could result in a stronger cell attachment of the virus, with both factors likely increasing viral transmissibility, infectivity, and pathogenicity.

## INTRODUCTION

The recent emergence of mutational variants of severe acute respiratory syndrome coronavirus 2 (SARS-CoV-2 [nCoV]) around the globe suggests adaptive evolution of the virus, potentially affecting its transmissibility, infectivity, virulence, and/or immune escape (1–4). The primary target of current vaccines and monoclonal antibodies is the spike protein, which mediates viral attachment to and entry into host cells (5, 6). Thus, the emergence of variants with mutations in the spike protein is of particular interest due to their potential for reduced susceptibility to neutralizing antibodies elicited by vaccination or prior infection. Nearly a dozen nCoV variants have been recently designated variants of concern (VOCs) or variants of interest (VOIs) and given Greek letter-based nomenclature (7).

The spike protein is 1,273 amino acids long and is composed of N-terminal region S1 (amino acids [aa] 14 to 682), responsible for viral attachment to target cells via the angiotensin-converting enzyme 2 (ACE2) receptor, and C-terminal region S2 (aa 686 to 1273), responsible for membrane fusion and cell entry (8). Before fusion, S1 is cleaved from S2 in the cleavage region (aa 682 to 685). Antibodies against the ACE2 binding domain of S1 (receptor-binding domain [RBD]; aa 319 to 541) are considered critical in neutralizing nCoV (9–11). Because of the important functional and antigenic properties of the RBD, structural changes in this domain deserve special attention and have already been highlighted by such notorious RBD mutations as E484K (e.g., found in B.1.1.28, also known as the “Brazil” variant or VOI zeta) and N501Y (found in B.1.1.7, also known as the “British” variant or VOC alpha, and in B.1.351, also known as the “South African” variant or VOC beta) (4).

We evaluated the feasibility of determining mutational changes in the S1 region by PCR amplification of a 541-base fragment within and immediately downstream from the RBD coding region (see Materials and Methods). This size of amplicon was chosen because it is possible to reliably determine its nucleotide sequence in a single run by standard Sanger sequencing. The amplicon included the gene region coding aa 414 to 583 of the spike protein (“region 414 to 583”), which includes the so-called receptor-binding ridge epitope (aa 417, 455, 456, and 470 to 490), the 443-to-450 loop epitope (aa 443 to 452 and 494 to 501) and the 570-to-572 loop of the so-called C-terminal domain 1 (CTD1) of S1, which is important in the interaction between the S1 and S2 regions. As test samples, we used 570 clinical oropharyngeal specimens collected during April and May 2020 from nCoV-positive patients at Kaiser Permanente Washington (51 samples) and nCoV-positive clinical samples submitted for testing to IEH Laboratories, Inc. (Bothell, WA) from different states during September 2020 to February 2021 (see Table S1 in the supplemental material). The latter were split into a September-October collection group (85 samples) and a November-February collection group (434 samples). In the process of region 414 to 583 analysis, we noted a high prevalence of samples that carried nCoV variants with an L452R mutation. This prompted us to perform an in-depth follow-up analysis of the L452R-carrying nCoV strains in our samples and a determination of their prevalence and clonal origin on a global scale using publicly available nCoV genomes and analytical tools of GISAID and Nextstrain databases.

## MATERIALS AND METHODS

### Sample collection.

Random deidentified nasopharyngeal samples were provided either as original swabs by Kaiser Permanente Washington (KPWA; April to May 2020, from the greater Seattle area) or as purified RNA by IEH Laboratories and Consulting Group (September 2020 to February 2021, from multiple states). Samples that tested positive for the presence of SARS-CoV-2 RNA (according to the respective laboratory practices, threshold cycle [*C_T_*] ≤ 32, *N* = 571) were subjected to further analysis. IEH samples of interest (see below) were assigned a unique identifier based on their collection date and source; information regarding the state of origin was provided for these samples for epidemiological analysis (see Table S2 in the supplemental material). All protected health information and personal identifiers were completely removed from all samples before they were provided for the study. The Western Institutional Review Board (Puyallup, WA) provided institutional biosafety committee services to IEH by approving consent forms and human research safety protocols.

### RNA isolation and SARS-CoV-2 testing.

RNA from KPWA samples was isolated using both the AllPrep DNA/RNA kit (Qiagen) and the MagMax viral pathogen RNA isolation kit (Thermo Fisher Scientific) according to the manufacturers’ procedures. RNA isolation from IEH samples was performed according to laboratory guidelines using a Thermo Fisher Kingfisher-96 instrument and proprietary IEH nucleic acid extraction reagent kit. RNA was stored at −20°C until use. Testing of RNA for the presence of SARS-CoV-2 RNA at KPWA and IEH was performed according to laboratory guidelines using the CDC 2019 novel coronavirus (2019-nCoV) real-time reverse transcriptase PCR (RT-PCR) diagnostic panel and a proprietary IEH SARS-CoV-2 RT-PCR test kit (based on the CDC RT-PCR kit), respectively.

### Amplification and sequencing of 414-to-583 and 1-to-250 regions of the spike protein.

To amplify either the 414-to-583 RBD region or the 1-to-250 region of the spike gene from SARS-CoV-2 RNA samples, we used both one-step RT-PCR and two-step (cDNA synthesis followed by separate PCR amplification) reaction designs with a commercial OneStep Ahead RT-PCR kit (Qiagen) or IEH in-house RT, PCR, and RT-PCR kits according to manufacturer’s guidelines. Both kits and conditions yielded identical results. All primers used for amplification are listed in Table S5. The first round of PCR (either RT-PCR on RNA or PCR on cDNA) consisted of 40 cycles of 10 s at 95°C, 15 s at 57°C, and 40 s at 72°C. The product was then diluted 1:50 with sterile water, and a second round of PCR was performed for 15 cycles under the same conditions by using T7-tailed nested primers to obtain a single pure product. Sanger sequencing of the amplified region was performed from both ends using standard T7Promoter and T7Terminator primers by Eton Bioscience, Inc. Sequences were analyzed using BioEdit 7.2 and MEGA 7 software. Sequences were compared to the reference Wuhan-Hu-1 sequence (NC_045512), and unique sequences were assigned allele numbers (see the supplemental alleles file).

### Whole-genome sequencing.

Whole-genome sequencing (WGS) was performed by IEH on a MiSeq Illumina instrument; each sample was subjected to two individual rounds of sequencing. Sequences were assembled *de novo* by using proprietary IEH pipeline or the PATRIC sequence assembly service (https://patricbrc.org/app/Assembly2). For detailed information on WGS, see the supplemental methods.

### Phylogenetic analysis of SARS-CoV-2 genomes.

The latest global analysis of SARS-CoV-2 genomes from the Nextstrain database (https://nextstrain.org/ncov/global) was used to determine viral genomes with the presence of L452 mutations in the spike protein (as of 12 June 2021). A.2.5, A.2.5.1, A.2.5.2, A.21, A.27, B.1.1.263, B.1.1.459, B.1.36, B.1.177.83, B.1.232, B.1.351, B.1.427, B.1.429, B.1.466, B.1.526.1, B.1.617.1, B.1.617.2, B.1.617.3, C.16, C.36.1, C.36.3, C.37, and P.4 were downloaded from the GISAID database (https://www.gisaid.org/, as of 12 June 2021) (see Table S4). Sequences were examined for mutations in position 452 of the spike protein. All L452R-containing sequences submitted in January and February 2021 for lineages B.1.232 (CAL.20A) and 50 randomly chosen B.1.427/B.1.429 (the epsilon variant) sequences were used to build phylogenetic trees based on all or only synonymous mutations by using MEGA 7 software. In addition, we used several closely related variants from both lineages without mutations in the L452 position (for the list of genomes used to build the tree, see Table S3). Deletions of stretches of nucleotides that resulted in in-frame codon deletions were treated as a single event. Unresolved nucleotides were assigned nucleotide value based on the closest relative. The same L452R-containing genome sequences for both lineages were used to calculate the average pairwise differences based on synonymous mutations.

### Statistical analysis.

The distributions of synonymous and missense mutations within the 414-to-583 fragment were analyzed for the RBD regions of interest (amino acids 417, 443 to 452, 455 to 456,470 to 490, 497 to 501, and 570 to 572; total, 49 amino acids) versus the remaining regions (total, 120 amino acids) by the McNemar test.

### Data availability.

All data are publicly available. Samples collected in this study and analyzed publicly available genomes metadata are listed in Tables S2 and S3, respectively. Primers used in this study are described in Table S5. Allele sequences for regions 1 and 2 are listed in the supplemental allele file. Viral whole-genome sequences were submitted to the GISAID database (accession numbers EPI_ISL_1081170, EPI_ISL_1081332, EPI_ISL_1081337, EPI_ISL_1081339, EPI_ISL_1081341, EPI_ISL_1081346, and EPI_ISL_1081347) (see Table S2).

## RESULTS

It was possible to amplify the 414-to-583 region and obtain a high-quality sequence by a single Sanger sequencing run for 570 of 571 (99.8%) specimens (1 sample failed to produce a PCR amplicon). Relative to the corresponding region of the reference Wuhan-Hu-1 genome (NC_045512), a total of 58 of the samples had 1 or 2 mutations. All mutations in the region were single nucleotide changes, i.e., point mutations. Among the April-May samples, only one sample (2.0% of the total) contained a mutation, which was of synonymous (“silent”) nature (c1425t) that did not result in an amino acid change (Table 1; see also Table S2 in the supplemental material). Among the September-October samples, four samples (4.7%) had a single mutation each: two different synonymous mutations (c1497t in two samples and t1695c in one sample) and one missense (nonsynonymous) mutation that resulted in the amino acid change E484K. Among the November-February samples, 53 (12.2%) had a total of 15 synonymous and 11 missense mutations, of which 14 samples were considered epidemiologically linked and thus duplicated. The linked samples were known to have been either isolated from the same patient or submitted from the same collection site on the same day, with all duplicated samples having the same mutational profiles. Upon removal of the duplicates, 39 of the epidemiologically nonlinked samples contained mutations in the 414-to-583 region, of which 27 had either unique single mutations or multiple mutations in unique combinations.

**TABLE 1:** Distribution of mutations across the test samples

Time period	No. of samples^*a*^	Site^*b*^	State	Region 1 (aa 414–583)^*c*^	Region 2 (aa 1–250)^*c*^
April–May (*N* = 51)	1	KP	WA	c1425t	Not done
September–October (*N* = 85)	1	22	CA	t1695c	
	1	4	CA	c1497t	
	1	10	CA	c1497t	
	1	23	PA	**g1450a (E484K)**	
November–February (*N* = 434)	1	3	CA	11290g	
	1	3	CA	t1326c	
	1	20	MN	t1338g	
	1	6	AZ	t1356g	
	1	4	CA	c1425t	
	1	UN	UN	t1458c	
	1	15	MN	t1506g	
	2	18	NM	c1524t	
	1	13	WA	c1626t	
	1	7	CO	c1770t	
	1	2	CA	**t1355g (L452R)**	**g38t (S13I) + g456t (W152C)**
	1	12	WA	**t1355g (L452R)**	**g38t (S13I) + g456t (W152C)**
	2	4	CA	**t1355g (L452R)**	**g38t (S13I) + g456t (W152C)**
	2	14	WA	**t1355g (L452R)**	**g38t (S13I) + g456t (W152C)**
	1	17	CA	**t1355g (L452R)**	**g38t (S13I) + g456t (W152C)**
	1	16	CA	**t1355g (L452R)**	**g38t (S13I) + g456t (W152C)**
	2	5	CA	**t1355g (L452R)**	**g38t (S13I) + g456t (W152C) + del141–144 LGVY**
	1	4	CA	**t1355g (L452R)**	**g38t (S13I) + g456t (W152C) + c76t**
	1	4	CA	**t1355g (L452R) + c1416t**	**g38t (S13I) + g456t (W152C)**
	2	8	WA	**t1355g (L452R) + t1593c**	**g38t (S13I) + g456t (W152C)**
	1	3	CA	**t1355g (L452R) + c1626t**	**g38t (S13I) + g456t (W152C)**
	2	5	CA	**t1355g (L452R)**	
	2	4	CA	**t1355g (L452R) + t1715g (T572I)**	
	1	21	GA	**g1365t (L455F)**	
	1	4	CA	**c1387t (P463S) + t1503c**	
	1	7	CO	**c1409a (T470N)**	
	5	4	CA	**c1433a (T478K)**	
	1	19	CA	**c1433a (T478K)**	
	1	10	CA	**c1433a (T478K) + c1686t**	
	1	1	MN	**g1445t (G482V)**	
	1	1	MN	**g1450a (E484K)**	
	2	20	MN	**g1450a (E484K)**	
	1	13	WA	**g1450a (E484K)**	
	1	9	WA	**t1480c (S494P)**	
	1	7	CO	**g1696a (G566S)**	
	2	9	WA	**c1709t (A570V)**	

a Total number of samples from same site but from different patients.

b Each number is a unique identifier of a site. KP, Kaiser Permanente Washington; UN, site is unknown.

c Amino acid changes are in boldface font.

**FIG 1:**
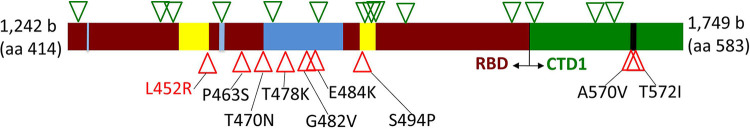
Distribution of silent (green triangles) and amino acid (red triangles) mutations across region 414 to 583 of the spike protein. Dark red, receptor-binding domain (RBD); green, C-terminal domain 1 (CTD1) of S1 spike region; blue, receptor-binding ridge epitope residues; yellow, 443-to-450 loop epitope residues; black, 570-to-572 loop residues.

Synonymous mutations were distributed across the 414-to-583 region, without clear clustering (Fig. 1). In contrast, the missense mutations were distributed nonrandomly and, except for one mutation (P463S), were clustered either within the main epitope regions of RBD or in the 570-to-572 loop of CTD1. Overall, there were 6 versus 1 missense mutation and 7 versus 8 synonymous mutations in RBD/CTD1-loop regions versus the remaining regions, respectively, resulting in a McNemar chi-square value of 4.50 (*P* = 0.034). All of the amino acid mutations had been reported previously and were identified in nCoV sequences deposited to GISAID, with five samples containing the common E484K mutation found, among other lineages, in the Brazil variant B.1.1.28 (VOI zeta). By far, however, the most frequent mutation found in region 414 to 583 was L452R, occurring in 14 of 39 (35.9%) nonduplicative samples and isolated from 9 of 18 separate collection sites.

L452R was found mostly but not exclusively in samples and sites from California. Indeed, the L452R mutation has received significant attention due to the report of the California Department of Public Health released 17 January 2021 and a follow-up publication (12) stating a sharp rise in isolation of nCoV variants with L452R across multiple outbreaks in California. According to the GISAID database, while only 6 nCoV genomes with L452R were deposited in September and October 2020 (all from California), 142 additional genomes with the mutation were deposited in November 2020 (95.7% from California), 488 were deposited in December 2020 (79.1% from California), and 619 were deposited in January 2021 (69.2% from California). This expansion has been linked to a single viral variant from PANGO lineage B.1.427/B.1.429, the Nextstrain clade 21C, which was originally designated CAL.20C and recently designated VOI epsilon (used here).

According to genomic analysis, the epsilon variant has also been defined by 4 additional amino acid mutations, including two spike protein mutations, S13I and W152C, located in the signal peptide and N-terminal domain, respectively (12). Using an approach similar to our sequencing of region 414 to 583, we amplified and sequenced the aa 1 to 250 coding region in all samples with the L452R mutation. Both S13I and W152C mutations were found in 10 nonduplicative samples, suggesting their identity with the epsilon variant. Surprisingly, in 4 samples with L452R, no additional mutations in the N-terminal domain were found. Those samples originated from two separate sites (two samples in each) in California, with one of the sample pairs carrying the T572I mutation in the 414-to-583 region (see above).

**FIG 2:**
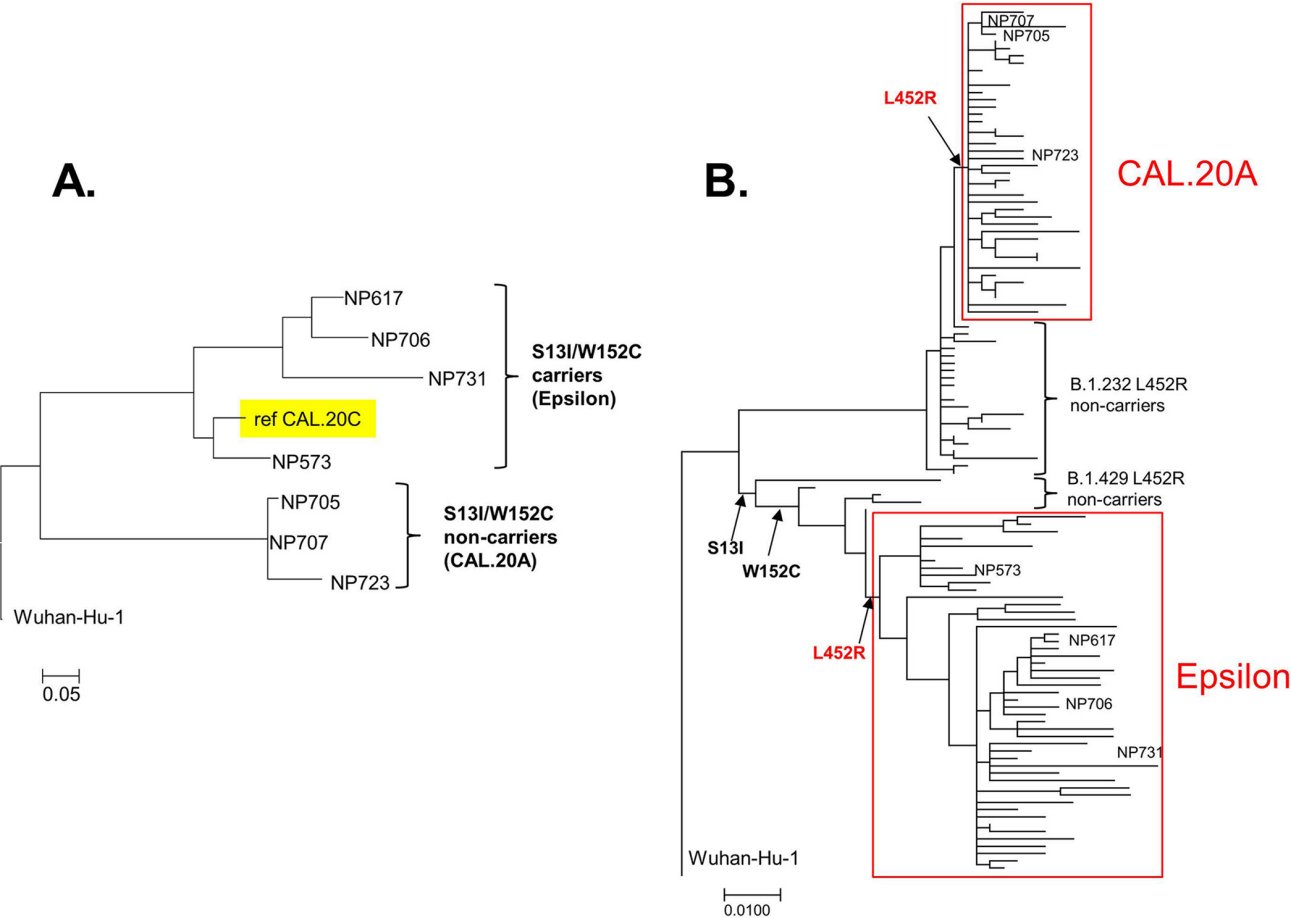
Phylogenetic trees of CAL.20A and the epsilon variant genomes. (A) nCoV strains identified in the tested samples. (B) nCoV strains deposited into GISAID database.

To determine how closely the L452R variants without S13I and W152C mutations were related to the epsilon variant, full-genome sequencing was performed on three of those samples and on four L452R samples with S13I and W152C. Based on the genome-wide analysis, all nCoV variants with L452R and S13I/W152C mutations were in the same clade as the reference epsilon variant strain (GISAID number 730092; isolated in September 2020) (Fig. 2A). In sharp contrast, the variants without S13I/W152C mutations formed a distinct phylogenetic clade that is distant from the epsilon variants and shared none of the epsilon variant-specific mutations. In fact, further analysis established that those strains derived not from the epsilon variant-containing clade 21C but from clade 20A, PANGO lineage B.1.232. We designated this novel L452R-carrying variant 20A/S:452R/B.1.232 (CAL.20A for brevity).

Analysis of the GISAID-deposited genomes revealed that, on 12 June 2021, the CAL.20A-including lineage B.1.232 contained a total of 1,494 deposited genomes, but only 453 of them (30.3%) contained the L452R mutation. The earliest deposition of an CAL.20A genome was made on 18 November 2020, from California (GISAID number 1794955), with 45 CAL.20A genomes deposited in November and December 2020, 248 deposited in January and February 2021, and 159 deposited in March and May 2021. The vast majority of CAL.20A strains were isolated in the states of California (50.9%) and Washington (37.3%) but also in 15 other states across the United States as well as Sweden, Mexico, Colombia, Canada, Norway, India, Costa Rica, England, and Denmark. Interestingly, one of the CAL.20A samples was derived from a gorilla in the San Diego Zoo (deposited to GISAID on 10 January 2021; GISAID number 862722)—a highly publicized case of nCoV infection in apes (13).

To compare the clonal diversity of CAL.20A and the epsilon variant strains, we used whole-genome sequences to build a phylogenetic tree of the January isolates of CAL.20A and 50 randomly selected genomes of the epsilon variant also deposited in January 2021 (Fig. 2B; Table S3). In addition, we included in the analysis some genomes that we have identified as the most closely related to the L452R variants but without this mutation. Based on the shorter branches overall, the CAL.20A cluster appeared to be significantly less diverse than the epsilon variant cluster. Indeed, the pairwise difference in the number of synonymous mutations, presumably neutrally accumulating 2.56 ± 1.41 and 8.22 ± 4.19 mutations per genome, respectively (*P* < 0.01), indicated that CAL.20A has emerged much more recently than the epsilon variant.

Relative to the Wuhan-Hu-1 reference strain and in contrast to the epsilon variant, L452R was the only omnipresent amino acid mutation in the spike protein of CAL.20A. Other mutations were found only sporadically, with most mutations in the S2 region. In fact, L452R was the only missense mutation in the entire genome that separated CAL.20A from the closest non-L452R strain within the B.1.232 lineage (GISAID number 636127) (Fig. 2B). Thus, acquisition of L452R appears to be the primary evolutionary event that triggered emergence of CAL.20A.

It was originally reported that, besides L452R, all epsilon variant strains carry 4 more missense mutations in the genome, including S13I and W152C in the spike protein. However, we found genomes in the B.1.427/B.1.429 lineage that are very closely related to the epsilon variant but are without L452R. At the same time, some of those strains carry either S13I alone (GISAID number 977963) or S13I together with W152 (GISAID number 847642 and number 977918) (Fig. 2B). Thus, the spike mutations in the epsilon variant-containing lineage were acquired sequentially, and the L452R mutation was acquired last. This suggests that, similar to that for CAL.20A, L452R was a trigger event for the massive clonal expansion of the epsilon variant.

**FIG 3:**
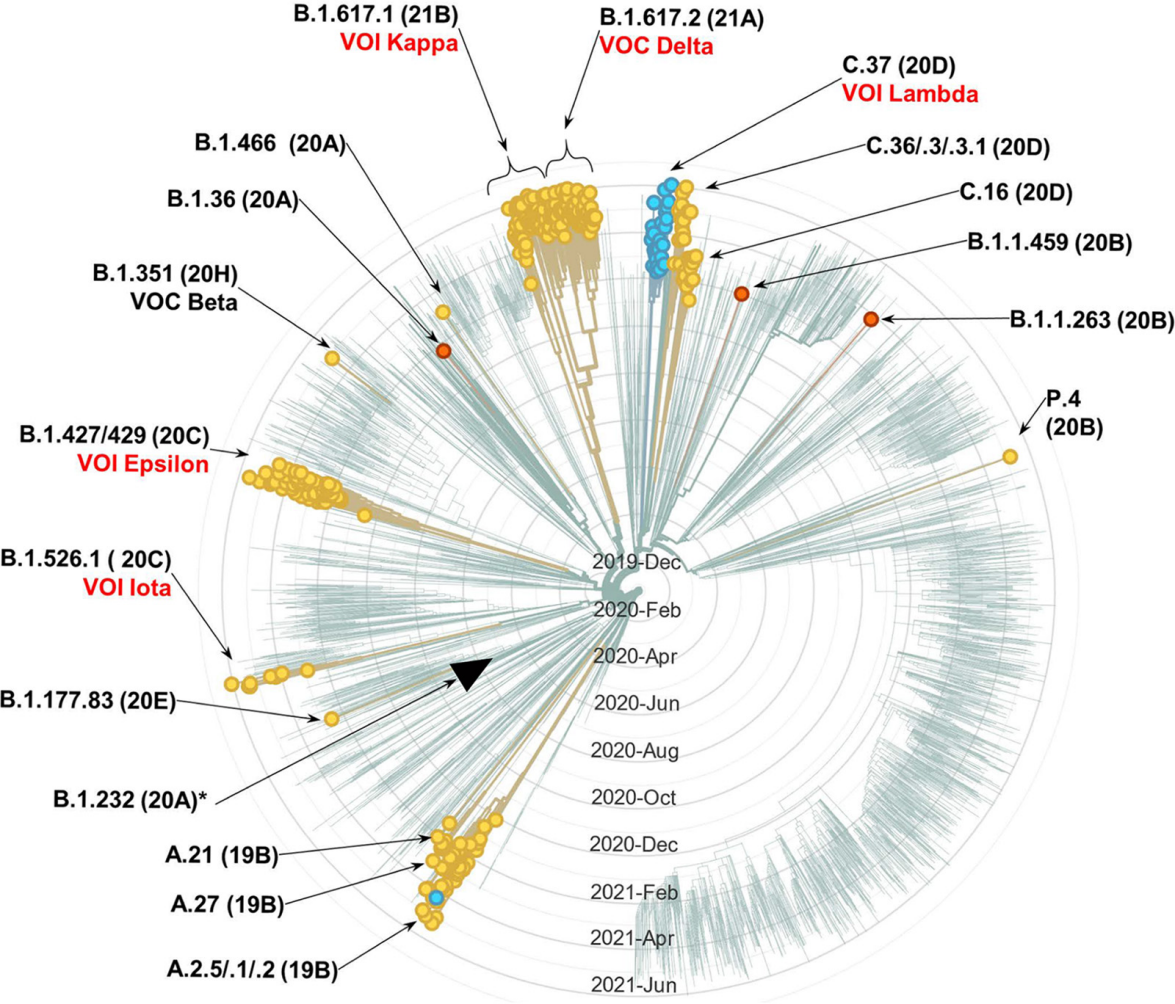
Nextstrain-generated radial cladogram of SARS-CoV-2 genetic variants (as of 12 June 2021). Showing 519 of 3,883 genomes randomly sampled between Jun 2020 and May 2021, belonging to 23 PANGO lineages. Circles tip branches containing genomes with any mutation in L452 (circles on branches without the mutations were manually removed for a visual clarity). Yellow circles, branches with L452R; blue, with L452Q; orange, with L452M. Black triangle tips the branch of lineage that includes CAL.20A. (At the date of analysis, CAL.20A strains were not among the sampled genomes in the Nextstrain database, and only three B.1.232 [all non-CAL.20A] lineages were found). Nomenclature: PANGO lineage followed by Nextstrain clade in parenthesis and the current VOC/VOI designation. In red, VOC/VOI with an omnipresent or nearly omnipresent L452 mutation (VOC beta is in black, as the L452 mutation is found only in few genomes of the variant).

Examination of the GISAID database on 12 June 2021 revealed a total of 60,273 nCoV genomes with L452R. Besides the epsilon variant and CAL.20A, at least 20 other separate PANGO lineages contained the mutation (Table S4). These lineages are from different clades, with the strains isolated in dozens of countries from all continents. A Nextstrain-generated phylogenetic tree of a subset of the lineages in which L452R occurs, based on genomes randomly sampled from GISAID by Nextstrain on the same day (i.e., 12 June 2021), is depicted in Fig. 3. Notably, the notorious “Indian” lineage B.1.617.2 (VOC the delta) contained L452R, as did its close cousin B.1.617.1 (VOI kappa). The delta and kappa variants differed from each other by the presence of additional mutations located in the 414-to-583 region of the RBD targeted for amplification in our study, i.e., T478K and E484Q, respectfully. Interestingly, a different missense mutation at position L452—L452Q—was found in one more epidemiologically important lineage (C.37; VOI lambda) that has been expanding in Peru and Chile and has an additional mutation in the 414-to-583 regio—F490S. Genomes with two other mutations at position L452—L452M and L452E—were also found, though they did not form as extensive lineages. There is a clear temporal trend in the number of isolated strains carrying the L452R mutations, with only 47 strains identified before November 2020, 2,594 identified in November and December 2020, 21,068 identified in January and February 2021, and 80,293 identified since March 2021 (Table S4). According to the GISAID genome depositions, in May 2021, L452R mutations were found in 15.9% of all nCoV isolates. In June 2021, 65.9% of all isolates had the mutation, primarily due to the massive expansion of the Delta variant.

## DISCUSSION

Taken together, our results show that in late 2020 to early 2021, two independent nCoV variants emerged in the state of California that carry the L452R mutation in the spike protein: the previously defined and broadly expanded epsilon variant (12) and the more recently emerged CAL.20A identified here. The fact that, according to the genome-wide analysis, emergence of both CAL.20A and the epsilon variant was triggered by the L452R mutation provides direct evidence for the adaptive significance of this mutation specifically and also creates a potential opportunity to isolate and functionally compare naturally occurring isogenic variants of nCoV with and without L452R.

It is possible that the lack of additional mutations in the spike protein of CAL.20A could be the reason it has not undergone as extensive expansion as the epsilon variant. This would indicate that other mutations in the epsilon variant, such as S13I and W152C in the N-terminal domain of the spike protein, might enhance the adaptive impact of L452R, i.e., that the genomic background of L452R plays a significant role as the target of positive selection. Similarly, the recently and vastly spread delta variant as well as the kappa and lambda variants also have additional mutations in the RBD region targeted by amplification in our study. The fact that mutations in L452 have been acquired by more than a dozen independent lineages across multiple countries and continents shows that L452 is a mutational hot spot due to strong positive selection. Interestingly, it appears that the selection for L452R became especially strong relatively recently. This possibly reflects adaptive evolution of the virus in response to either the epidemiological containment measures extensively introduced in the fall of 2020 or a growing proportion of the population with immunity to the original viral variants, i.e., reconvalescent and vaccinated individuals. Thus, positive selection could favor increased transmissibility, infectivity, and/or immune escape of the virus. However, it is also possible that the delay in spread of L452 mutants until recently could be due to constraints on the fixation of advantageous mutations that are imposed by stochastic evolutionary events, such as bottlenecks, etc.

Though potentially an accidental event, isolation of CAL.20A from a gorilla at the San Diego Zoo is worthy of note. According to the sequence deposited in GISAID, the gorilla CAL.20A variant carries two additional point mutations, both in the ORF1ab nonstructural protein 2 (nsp2): a missense c810t (T183I) and synonymous c934t (unfortunately, several sequence stretches are missing in the genome). It is impossible to say at this point to what extent the isolation of the CAL.20A variant is connected to possibly distinctive biological properties of the strain and, specifically, the L452R mutation. However, it would be valuable to determine whether the occurrence of CAL.20A infection in the gorilla was due to specific features of CAL.20A with regard to viral virulence.

Several recent studies investigated the potential effects of L452R and other mutations. It was found that L452R reduces multifold the spike protein reactivity with viral neutralizing antibodies and sera from convalescent patients (9, 14–16). In particular, one study has shown that the L452R mutation reduced neutralizing activity of 14 of 34 RBD-specific monoclonal antibodies (16). Interestingly, the same study showed that the S13I and W152C mutations that are present in the S protein of the California-dominating B.1.427/B.1.429 lineage, but absent in CAL.20A, resulted in total loss of neutralization by all 10 N-terminal domain (NTD)-specific monoclonal antibodies tested. This supports the hypothesis that mutations in position L452 in combination with other missense mutations in S1 are likely to have a cumulative immune escape effect. Furthermore, it was shown that L452R confers viral escape from human leukocyte antigen (HLA)-restricted cellular immunity mediated by cytotoxic T lymphocytes (17). Finally, it was predicted that mutations in residue L452, which is located in immediate proximity to the RBD-ACE2 interaction interface, result in at least a modestly higher affinity of receptor binding and thus an increased rate of human cell infectivity (17–19). Both the successful immune escape and strengthened virus-cell attachment of L452 mutants might lead to increased transmissibility, infectivity, and/or pathogenicity of nCoV.

We cannot exclude the possibility of sample collection bias in our study, as it was not originally designed as an in-depth surveillance study in specific geographical regions. In addition, our analysis is limited to a relatively small set of samples in hand and to publicly available genomes. However, we believe that the identification of CAL.20A and other nCoV variants with mutations at position L452 will be useful in the optimization of real-time monitoring and a more complete understanding of the biological properties of this pandemic virus with a recently expanding number of genetic variations that are cause for significant public concern.
