# Supplementary material for: Acquisition of the L452R Mutation in the ACE2-Binding Interface of Spike Protein Triggers Recent Massive Expansion of SARS-CoV-2 Variants
Source: J Clin Microbiol. 2021 Oct 19;59(11):e00921-21. doi: 10.1128/JCM.00921-21 (PMC8525575; doi:10.1128/JCM.00921-21)
Supplement: Supplemental file 6 — Supplemental methods. Download JCM.00921-21-s0006.pdf, PDF file, 0.1 MB [file jcm.00921-21-s0006.pdf]

Genome Sequencing

Library preparation for genome sequencing was done using COVID-19 ARTIC v3 Illumina library construction and sequencing protocol V.4 as described below (https://www.protocols.io/view/covid-19-artic-v3-illumina-library-construction-an-bgxjjxkn?version_warning=no).

For cDNA synthesis, 4µL LunaScript Super Mix (NEB, Ipswich, MA, USA) was added to 6ul Nuclease-free water and 10 µL extracted RNA in a PCR tube followed by incubation in the thermal cycler with the following conditions 25 °C for 2 min, 55 °C for 20 min, and 95 °C for 1 min. The synthesized cDNA (8.8 ul each) was transferred to two new PCR tubes (Tube #1 and Tube #2). 12.5 µL Q5 High-Fidelity 2X Master Mix (NEB, Ipswich, MA, USA) was added to Tube #1 and Tube #2. 3.7 ul of 10uM pool #1 primer mix was added to Tube #1, similarly pool #2 primer mix was added to Tube #2. PCR cycling was then performed as follows: 98 °C for 30 sec followed by 36 cycles of 98 °C for 15 sec and 65 °C for 5 min.

The resulting PCR products were combined and purified using PCRClean DX (Aline Biosciences, Woburn, MA, USA) at 1.8x bead to amplicon ratio and eluted in 30 µL of nuclease free water. Two µL of PCR product was quantified using a Qubit dsDNA HS (Thermo Fisher Scientific, Waltham, MA, USA) following the manufacturer's instructions. Illumina sequencing libraries were prepared using NEBNext Ultra II DNA Library Prep Kit (NEB, Ipswich, MA, USA) as follows. For end repair reaction, 200 ng of purified PCR product from the previous step was mixed with 2.8ul of NEBNext Ultra II prep buffer and 1.2 ul of NEBNext Ultra II prep enzyme in a PCR tube to a total volume of 24ul by adding Nuclease-free water. The reaction mixture was then incubated in the thermal cycler at 20 °C for 30 min followed by 65 °C for 20 min. The end repaired DNA (24ul) was then added to a PCR tube containing 5ul of 15uM Universal TruSeq Adapter (IDT, Coralville, IA, USA), 0.6ul of NEBNext Ligation enhancer, and 18ul of NEBNext Ultra II Ligation Master Mix followed by incubation at 20 °C for 15 minutes. The resulting adapter ligated library was purified using PCRClean DX at 0.8X bead ratio to library and eluted in 25ul of nuclease free water. Two ul of library was quantified using Qubit dsDNA HS kit.

The adapter-ligated DNA was amplified using KAPA HiFi HotStart ReadyMix (Roche, Basel, Switzerland) as follows. 100 ng of adapter ligated DNA fragments were added to a PCR tube containing 5ul of 10uM IDT 8 CDI index primer pair (IDT, Coralville, IA, USA) and 25 ul of KAPA HiFi HotStart ReadyMix (2X) to a total volume of 50ul by adding Nuclease-free water. PCR cycling was then performed as follows: 95 °C for 5 minutes followed by 4 cycles of 98 °C for 30 sec, 65 °C for 30 sec,72 °C for 2 min and a final extension at 72 °C for 5 min. The resulting library was purified using PCRClean DX at 0.8X bead ratio to library and eluted in 50ul of 10mM Tris-Hcl pH8.0. Two ul of library was quantified using Qubit dsDNA HS kit.

The genome sequencing was performed using the Illumina MiSeq desktop sequencer (Illumina, San Diego, CA, USA) loaded with a paired-end 2 × 250 cycle MiSeq reagent kit version 3. The raw shotgun reads were then quality filtered using fastp software version 0.20.0 and then assembled denovo using MEGAHIT assembler version v1.2.9.

References:

Shifu Chen, Yanqing Zhou, Yaru Chen, Jia Gu; fastp: an ultra-fast all-in-one FASTQ preprocessor, Bioinformatics, Volume 34, Issue 17, 1 September 2018, Pages i884–i890, https://doi.org/10.1093/bioinformatics/bty560

Li, D., Liu, C-M., Luo, R., Sadakane, K., and Lam, T-W., (2015) MEGAHIT: An ultra-fast single-node solution for large and complex metagenomics assembly via succinct de Bruijn graph. Bioinformatics, doi: 10.1093/bioinformatics/btv033 [PMID: 25609793].

Li, D., Luo, R., Liu, C.M., Leung, C.M., Ting, H.F., Sadakane, K., Yamashita, H. and Lam, T.W., 2016. MEGAHIT v1.0: A Fast and Scalable Metagenome Assembler driven by Advanced Methodologies and Community Practices. Methods.
